# Use of ash filtrate as an alternative to chemical disinfectant and its antimicrobial efficacy

**DOI:** 10.5455/javar.2024.k851

**Published:** 2024-12-28

**Authors:** Khairun Nahar Shithi, Ananna Saha, Md. Nurul Haque, Ismail Hossain, Mohammed Nooruzzaman, Jahan Ara Begum, Rokshana Parvin, Emdadul Haque Chowdhury

**Affiliations:** 1 Department of Pathology, Faculty of Veterinary Science, Bangladesh Agricultural University, Mymensingh, Bangladesh; 2 Bangladesh Livestock Research Institute, Savar, Bangladesh

**Keywords:** Household ash, hand-made filtration system, ash filtrate (AF), antimicrobial efficacy, disinfectant, sanitizer

## Abstract

**Objective:**

The study aims to prepare the ash filtrate (AF) from household ashes using an in-house-designed handmade filtration system and to observe the antimicrobial efficacy and sanitizing effects.

**Materials and Methods:**

Household ashes from various plant sources were collected, and AF was prepared through a handmade filtration system after adding water. The pH of stock AF was measured, and 20%, 40%, 60%, and 80% AF solutions were prepared by adding distilled water in appropriate proportions to obtain a wide range of pH values. The antimicrobial efficacy of AF against *Salmonella* spp. *in vitro*, Newcastle disease virus (NDV), and low pathogenic avian influenza virus (LPAIV) H_9_N_2_ *in ovo* were analyzed. Contaminated eggs were individually sprayed or dipped with AF to detect the bacterial load on the eggshell surface. Further experimental use of AF as an egg sanitizer in routine biosecurity operations in broiler sheds was also evaluated.

**Results:**

The prepared AF showed high alkalinity; pH varied from 10.7 to 8.20 and contained a higher amount of K, Na, and Cl. The alkaline AF and its dilution gradually inhibited Salmonella growth and showed gradual pH-dependent antibacterial efficacy. Similarly, AF and its dilution showed a gradual decrease in viral titer against the LPAIV (H9N2); however, antiviral activity against the velogenic strain of NDV was quite steady. Applying AF as an egg sanitizer also reduced the bacterial loads significantly on the eggshell surface compared to untreated eggs. Moreover, AF having pH 10.5 experimentally used in routine sanitization practices of a boiler shed resulted in low bird mortality (10/210), higher body weight gain, and a low feed conversion ratio compared to the untreated control flock.

**Conclusion:**

The higher alkalinity of the AF is responsible for the antimicrobial activity of commercial disinfectants. Consequently, we can use AF as a low-cost, effective, natural antimicrobial agent to replace chemical disinfectants.

## Introduction

The poultry sector of Bangladesh is contributing to the nation with a potent source of animal protein through poultry eggs and meat. The annual egg production is 23.37 billion, which has already exceeded the immediate demand of 104 eggs per person per year, where the average production is 134.58 eggs per person per year [1]. This was made possible by the early spread of backyard, small-scale, and commercial poultry farming, which was primarily spearheaded by stakeholders, young businesspeople, and rural women who were able to support themselves financially [2,3], addressing the fact that the poultry industry has gained significant traction [4].

However, infectious disease outbreaks are a major hindrance to the continuous progress of the poultry industry [5]. Among these, the most significant endemic-circulated infectious diseases in poultry are *Salmonella *infections, Newcastle disease, and Avian Influenza [2,6]. Incidences of these diseases were found at 14.29%, 17.2%, and 25.5% in different studies, which caused both production losses and economic disaster for the farmers [6,7].

Poor biosecurity is observed in middle-to-small-scale poultry farms, resulting in high disease risk in both poultry and human health [8]. A high level of farm biosecurity, along with proper cleaning and disinfection, is crucial to control the disease with the complete elimination of pathogens. Usually, disinfectants used in farm biosecurity practice are mostly chemical bases, including quaternary ammonium, chlorine, iodine, aldehyde, and alcoholic compounds [8]. However, the microorganisms very often get resistant to commonly used disinfectants [9]. Uncontrolled antimicrobial uses, particularly in farming systems accelerate the risk of pathogenic microbes producing antimicrobial resistance (AMR) genes that are transmitted from livestock to humans via the food chain [10] and these AMR organisms showed decreased disinfectant susceptibility [11].

Table eggshells that are contaminated are a major source of foodborne Salmonellosis, which is a serious public health issue [12]. Eggshells become contaminated by horizontal transmission from an infected cloaca during laying or from contaminated external environments such as faces, feed, water, feeding utensils, personnel handling, and fomites [13,14]. Various chemicals disinfected repeatedly used in sanitizing eggs generate residue in the food chain, resulting in the current threat of AMR as well as developing disinfectant resistance [12].

Because of such concern, an alternative to the chemical disinfectant, a safe and natural source compound with verified antimicrobial activity, is highly required both for the sanitization of the egg as well as in routine biosecurity practices in poultry farming operations, such as cleaning of equipment, spraying surrounding the farm premise, in litter treatment, in a foot bath, and hand washing after handling birds to reduce the microbial burden in the farming system. From ancient times, rural people have been using household-derived ashes for their various day-to-day purposes, including cloth and dishwashing, brushing teeth, washing hands after defecation, sawing surrounding areas of latrine, as well as applying on faces as a desiccant and odor absorbent, and also in the agricultural field to control the pest.

Wood ash consists of both inorganic and organic residue produced after the combustion of wood, wood products, and tree or woody plant-derived parts such as leaves, twigs, branches, stems, roots, and bark [15]. This ash is an alkaline substance, previously used as a source of potash [16], as fertilizer and liming material to maintain the soil pH [17], used in making local soap from ash-derived alkali and sanitizing the fecal sludge [18–20]. Furthermore, wood ash extract used in neutralizing the high tannin in sorghum used in poultry feed resulted in increased growth of poultry [21].

The World Health Organization suggested ash as an alternative to soap [22]. Ash filtrate (AF) can be used in place of chemical disinfectants as a low-income, small-scale farmer’s natural, safe, and cost-effective sanitizer. Therefore, the study was designed to prepare the AF from household-derived ashes with different plant origins, along with an estimation of their pH with chemical analysis of pooled AF, investigation of the antibacterial and antiviral efficacy against *Salmonella* spp. isolated from table eggshell surface samples *in vitro*, and Newcastle disease virus (NDV) and low pathogenic Avian Influenza virus (LPAIV) H9N2 in the* in-ovo* system, respectively. In addition, an experimental trial of AF was carried out in sanitizing eggshells and poultry farm biosecurity practices as an alternative to chemical disinfectants.

## Materials and Methods

### Ethical approval

The current study has been approved by the ethical committee of the Bangladesh Agricultural University Research System under the approval number BAURES/ESRC/46/2024.

### Preparation and upgradation of AF from household ashes

**Figure 1.**
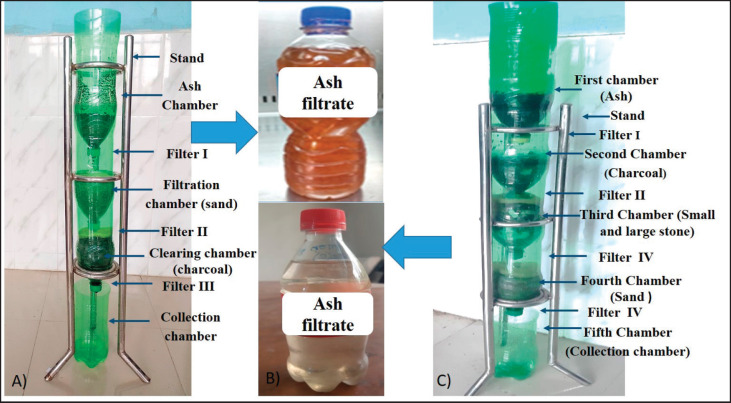
Handmade filtration system and the physical properties of AF. A) Handmade filtration system that consists of four chambers. B) Straw to brown color to clear AF using a handmade filtration system. C) Upgraded handmade filtration system that consists of five chambers.

First, 20 household-derived ashes from different plant sources were collected from selected areas of the Mymensingh, Rangpur, and Dinajpur districts. A handmade filtration system was developed and used to filtrate ash samples. First, the filtration system consisted of four chambers: a) an ash chamber for sampled ash; b) a filtration unit containing sand; c) a clearing unit containing charcoal; and d) a collection chamber for reserving the AF. A thin layer of foam was used as a filter in the bottom of the first three chambers in association with the sand and coal layer at the top as an additional filter (Fig. 1A). However, to make the AF clearer and to remove the color, the upgraded version was introduced, including a chamber containing small and large stones, along with changing the chamber arrangement, which was structured into five chambers: I) first chamber for ash; II) second chamber for charcoal; III) third chamber for small and large stones; IV) fourth chamber for sand; and V) fifth chamber for AF. Additionally, a foam layer with cotton was used over and below the second, third, and fourth chambers to enhance the filtration process (Fig. 1C). Filtration of the ash samples was done by adding 500 ml of water with 100 gm of each ash sample through the developed filtration system overnight (12 h). After filtration, AF was settled down in the collecting chamber.

### Physical and chemical analysis of the AF

In the developed handmade filtration system, 20 ash samples were filtered separately, pH was measured with a pH meter (Mettler Toledo, USA), and the AF was stored for 6 months to evaluate their pH stability. A part of all AF was pooled, and the pH of the pooled AF was 10.5, which was sent for chemical analysis. The mineral constituent of the pooled AF was analyzed using the following methods—Na & K: flame emission spectrophotometric method, Ca and Mg: EDTA titrimetric method, S: turbidimetric method, P: spectrophotometric method, CO_3_ and HCO_3_: titrimetric method, and Cl: argentometric method of titration.

### Isolation of Salmonella spp. from table eggshell surfaces

Antibacterial efficacy of AF was performed after the isolation of *Salmonella *spp. from table eggshell surface samples. A total of 100 table eggshell surface samples were collected using sterile cotton swabs moistened with sterile phosphate buffer solution (PBS). The culture of the samples was done according to standard guidelines with some modifications [23,24]. Briefly, enrichment of individual samples into nutrient broth (NB) aerobically at 37°C for 24 h, streaking on nutrient agar plates, and subculturing onto selective agar such as *Salmonella*–*Shigella* (SS) agar, xylose lysine deoxycholate (XLD agar), and brilliant green agar (BGA) several times up to obtaining a pure colony. Identification of suspected *Salmonella* spp. was confirmed phenotypically by colony characteristics, Gram staining, and biochemical tests: basic sugar fermentation test, methyl red (MR)-Voges–Proskauer test (VP), indole test, urease test, and triple sugar iron (TSI) slant test. Molecular confirmation was done by polymerase chain reactions (PCR) using gene-specific oligonucleotide primer sequences, F: 5’-ACT GGC GTT ATC CCT TTC TCT GGT G-3’ and R: 5’-ATG TTG TCC TGC CCC TGG TAA GAG A-3’ with the amplicon size of 496 bp that amplifies the members of the genus *Salmonella*. The DNA templates were extracted by boiling method. For PCR analysis, the reaction was carried out in a final volume of 25 μl containing 12.5 μl of master mix (Dream *Taq* 2X PCR Master Mix, Thermo Scientific, USA), 2 μl (10 pmol/μl) of each primer, R primer = 2 μl, 5 μl DNA template, and 3.5 μl of nuclease-free water. Electrophoresis of the PCR-amplified product was done by preparing 1.5% agarose gel with TBE buffer and finally visualized on a UV transilluminator.

### Experimental procedure of antibacterial efficacy of AF

The antibacterial efficacy of the AF was tested against one of the previously isolated *Salmonella *spp. First, undiluted AF (100%) with pH 10.5 was diluted into 20%, 40%, 60%, and 80% of AF solution by adding distilled water (DW) in appropriate proportions to obtain a wide range of pH values. The pH of these diluted AF was measured and used as treatments, whereas washing soda and sodium hydroxide with pH >11 were used as positive controls, and NB at pH 7.18 and DW at pH 7.11 were used as the negative control. The 10-µl *Salmonella *culture (2.58 × 10^8^ CFU/ml) was individually added into 5 ml of each 20%, 40%, 60%, 80%, and 100% AF solution and control groups and was mixed properly and then was incubated for 5 min at room temperature. One µl from each mixer was spread on a BGA plate (five plates for each mixture) and was incubated for 24 h at 37°C. Following the incubation, an average number of bacterial colonies were counted in five plates for each mixture and expressed as colony-forming units per milliliter (CFU/ml).

### Experimental use of AF as egg sanitizer

An AF of pH of 10.5 was selected for this study. First, five groups were made containing three eggs in each group: A) untreated eggs, B) AF sprayed, C) AF dipped, D) water sprayed, and E) water dipped. To detect the bacterial load on the eggshell surface, untreated eggs (group A) were dipped directly into 100 ml of sterile PBS in a beaker, whereas groups B and C were sprayed and dipped with 100% AF, respectively. However, group D and group E eggs were sprayed and dipped with DW at pH 7.11, respectively. Then, all treatment groups of eggs were individually dipped into 100 ml PBS to detect the bacterial load in eggshell-washed PBS. Then, 20 µl of eggshell-washed PBS from all groups was spread individually in three BGA plates and incubated at 37ºC for 48 h. The bacterial colonies were observed, and the average number of colonies on three plates for each group was calculated and finally recorded as colony-forming units per milliliter (CFU/ml).

### Experimental procedure of antiviral efficacy of AF in ovo

NDV with 2.5 X106 EID50 and LPAIV (LPAIVH9N2) with 5 × 10^5^ EID50 viruses were used for the antiviral efficacy testing of AF; 250 µl of undiluted (100%) AF with pH 10.5 and its 20%, 40%, 60%, and 80% dilutions were sprayed individually over 100 µl of each NDV and LPAIV H9N2 virus on Petri dishes, respectively, and incubated for 1 h at room temperature. The 100-µl AF-treated mixture from the respective Petri dishes was further mixed with 900 µl of media (MEM), and subsequently, 200 µl was inoculated in 9-day-old embryonated chicken eggs (three eggs for each AF) via the allantoic cavity route and incubated at 37oC for 72 h. Sodium hydroxide (pH 11.47), washing soda (pH 11), and DW (pH 7.11) are used as controls against the viruses, where high pH should inhibit complete growth and low pH should not have any effect on virus growth. Embryo changes were observed daily, and the hemagglutination test (HA) was performed in the harvested allantoic fluid according to standard procedure [25,26]. HA titer was calculated and correlated with the viral titer at different dilutions of AF and compared with the controls.

### Experimental use of AF as sanitizer in farm biosecurity practice

For this experiment, 420 broiler birds were reared in two groups (G1 and G2) in the experimental shed. Each group contains 210 birds with a sufficient supply of feed and water. In group G1, undiluted (100%) AF with pH 10.5 was used as a sanitizer in every management procedure, such as hand washing, cleaning of feeding equipment, and extensive regular spraying in and around the shed, whereas group G2 was treated similarly with normal tap water. Birds were reared from day-old chicks to 35 days and monitored daily. The body weight gain, feed conversion rate, and mortality rate of the two groups were compared and evaluated.

### Statistical analysis

Statistical differences in colony counts between various treatment groups were calculated using the Kruskal–Wallis test with Dunn’s multiple comparison test. *p*-values of ≤0.05 were considered statistically significant. The analyses were conducted using GraphPad Prism 5.0.

## Results

### Physical appearances and pH of the AF

Clear, straw-to-brown color and soapy appearance AF was found using a developed handmade filtration system (Fig. 1B). The pH values of the AF are between 10.7 (rice straw) and 8.2 (potato straw). A pH value of more than 10 was found in the case of five AFs. By adding 500 ml of water with 100 g of ash sample, an approximate range from 380- to 280-ml AF was obtained using the filtration system (Table 1). Moreover, after storing for 6 months, all the AF showed slight changes (±0.5) in the pH values, suggesting a stable alkaline nature of AF.

### Chemical constituents of the pooled AF

Composition analysis of the pooled AF revealed a very high concentration of potassium (K), chlorine (Cl), sodium (Na), and sulfur (S). Two important components, HCO_3_- and CO_3_ were found at moderate concentrations. Other minerals, including calcium, magnesium, and phosphorus, were found at minimum concentrations (Table 2).

### Salmonella spp. isolation and identification from table eggshell surface samples

**Figure 2.**
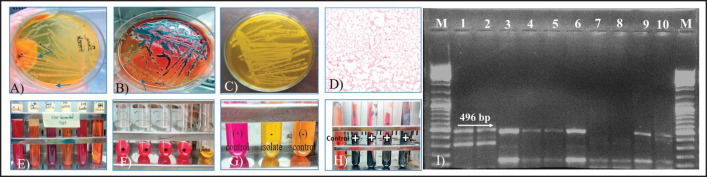
*Salmonella* colonies on A) SS agar, B) on BGA agar C) on XLD agar, D) pink color rod shape organism in Gram staining, (E) sugar fermented test, (F) MR test, (G) urease test, (H) TSI slant test, and (I) gel documentation image showing amplification of 496-bp fragment size positive for *Salmonella* genus. Lane: 1–6, 9, and 10 showing positive amplification. M: 100-bp DNA ladder.

*Salmonella* spp. was identified in the case of an 11% sample (11/100) based on culture characteristics, colony morphology, biochemical tests, and molecularly by PCR. All 11 positive isolates exhibited uniform turbidity in the NB, whitish colonies on nutrient agar, transparent round colonies with or without black centers on SS agar (Fig. 2A), whitish or cream-colored colonies on BGA agar (Fig. 2B), and black-centered colonies on XLD agar (Fig. 2C). Microscopically, Gram-negative, pink-colored, scattered arranged, and rod-shaped organisms were found in Gram’s staining (Fig. 2D). Fermented glucose, dextrose, and maltose with the production of acid and gas but did not ferment lactose and sucrose (Fig. 2E); MR positive (Fig. 2F); VP, indole, and urease (Fig. 2G) negative; TSI slant positive (Fig. 2H); and finally molecularly confirmed *Salmonella *spp. with genus-specific primer by PCR targeting the 496-bp amplicon size (Fig. 2I).

**Table 1.** pH of AF from household ashes using a developed hand-made filtration system.

Plant sources	p^H^	AF	Plant sources	p^H^	AF
1. Rice Straw	10.70–10.40	340 ml	11. Mulberry	09.40–09.20	330 ml
2. Plum wood	10.30–10.10	370 ml	12. Maize Straw	09.00–08.98	335 ml
3. Neem wood	10.20–10.00	350 ml	13. Eucalyptus wood	09.00–08.50	350 ml
4. Mahogany wood	10.06–09.80	335 ml	14. Bamboo root	09.20–08.80	330 ml
5. Mango wood	10.00–09.60	365 ml	15. Cow dung	08.80–08.50	280 ml
6. Tobacco root	09.98–09.80	290 ml	16. Rice husk	08.80–08.40	300 ml
7. Banana steam	09.86–09.50	380 ml	17. Wheat straw	08.60–08.50	320 ml
8. Bamboo	09.80–09.50	300 ml	18. Charcoal	08.60–08.50	280 ml
9. Mixed wood	09.70–09.60	345 ml	19. Mustard straw	08.40–08.20	300 ml
10. Mahogany leaf	09.60–09.50	310 ml	20. Potato straw	08.30–08.20	305 ml

**Table 2.** Mineral concentration of the pooled AF solution at pH 10.5.

Number	Components	Mineral concentration (mg/l)
1	K	32,172.73
2	Cl	13,495.82
3	Na	6,356.57
4	S	7,620
5	HCO3-	275
6	CO3	222
7	P	118.59
8	Mg	63.193
9	Ca	48.00

### Antibacterial efficacy of AF

**Figure 3.**
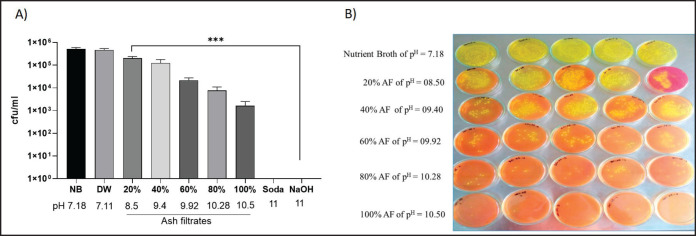
Antibacterial efficacy of AF in bacterial culture. A) AF of pH 10.5 with its 80% dilution (pH 10.28) and washing soda showed a significant decrease in the CFU counts, compared to NB and DW solutions. Data indicate mean ± SEM of 5 independent trials. The Kruskal–Wallis test with DMCT, *p ≤ 0.05, **p ≤ 0.01, and ***p ≤ 0.001. B) The bacterial growth on the agar plate gradually decreases with ascending dilution of AF, suggesting that AF inhibits bacterial growth.

After the addition of 10 µl with 2.58 × 10⁸ CFU/ml *Salmonella* culture into two negative controls, the number of colonies was 5.04 × 10^5^ CFU/ml and 4.65 × 10^5^ CFU/ml in the case of NB (pH 7.18) and DW (pH 7.11), respectively, whereas 100% AF with pH 10.5 and its 40%, 60%, and 80% dilutions with pH ranging from 10.5 to 8.50 showed gradual inhibition of bacterial growth (Fig. 3). Both the positive control washing soda and sodium hydroxide with pH >11.0 completely inhibited the growth of *Salmonella*. Moreover, significantly lower colonies (1.60 × 10^3^ CFU/ml) were observed in the case of 100% AF with pH 10.5 compared to NB and DW, indicating significant inhibition of *Salmonella* growth (Table 3 and Fig. 3A and B).

### AF as egg sanitizer

**Figure 4.**
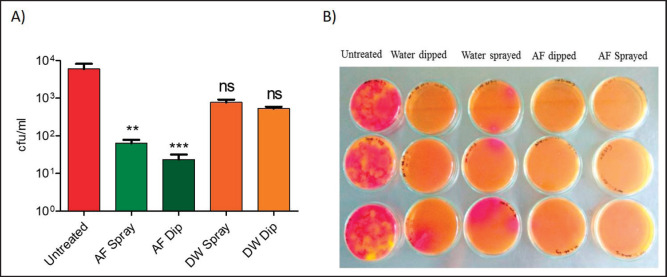
Antibacterial efficacy of AF after sanitization at the eggshell surface. (A) Bacterial loads in eggshell surface washes following treatment with AF and DW. Data indicate the mean ± SEM of 5 independent trials. Kruskal–Wallis test with DMCT, *p ≤ 0.05, **p ≤ 0.01, and ***p ≤ 0.001. (B) Bacterial loads in eggshell washes after treatment with AF and DW.

The average bacterial load in five sets of untreated, AF sprayed, AF dipped, DW sprayed, and DW dipped was 6 × 10^3^, 0.63 × 102, 0.23 × 102, 0.77 × 10^3^, and 0.53 × 10^3^ CFU/ml, respectively. Comparative analysis between different treatment groups showed that AF-sprayed and dipped eggs reduced significant bacterial loads on eggshell surfaces compared to the untreated eggs. Interestingly, treating eggs with DW also showed some reduction in bacterial loads (Fig. 4A and B).

### AF as an antiviral agent against LPAIV H9N2 and velogenic NDV

**Figure 5.**
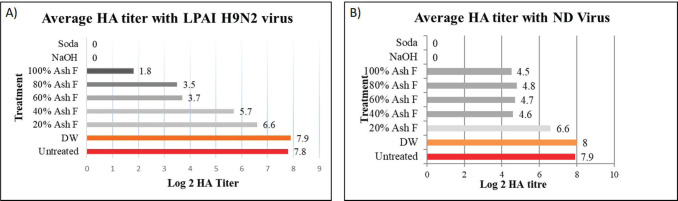
Antiviral efficacy of AF *in vivo* after inoculation with LPAIV and NDV. Column graphs showing average HA titer obtained following inoculation (3 replicate) of A) LPAIV (H9N2) and B) NDV, after being treated with different dilutions of AF and controls. Washing soda and NaOH-treated virus did not show any HA titer, whereas untreated or treated with DW showed expected viral growth.

Gradual reduction of HA titer was observed for LPAIV H9N2 treated with 100% AF and its different dilutions. The 100% AF-treated LPAIV showed the lowest average HA titer of 1.8 log 2; 60% and 80% AF achieved HA titers of 3.7 log 2 and 3.5 log 2, respectively, whereas 40% and 20% AF-treated LPAIV showed HA titers of 5.7 log 2 and 6.6 log 2, respectively (Fig. 5A). On the other hand, no significant gradual reduction of viral titer was observed between the different concentrations of AF at pH 10.5 and its mentioned dilutions sprayed on the NDV (Fig. 5B). An almost steady HA titer was exhibited for all treated dilutions of AF treated with NDV. However, significant HA titer was observed for both LPAIV and NDV when untreated or treated with DW. In contrast, when NaOH and washing soda (pH >11) were used, all embryos died without achieving any viral titer, indicating pH-dependent early embryo mortality.

**Table 3.** Summary of CFU count in AF of pH 10.5 with its different dilution as well as in control groups.

	Negative control		Trial with AF at pH 10.5 and its dilutions					Positive control	
	NB	DW	20%	40%	60%	80%	100%	Washing soda	Sodium hydroxide (0.1 M)
pH	7.18	7.11	08.50	09.40	10.18	10.28	10.5	11	11
CFU/ml	5.04 × 105	4.65 × 105	2.02 × 105	1.23 × 105	2.20 × 104	7.60 × 103	1.60 × 103	0	0

### AF as a sanitizer in the farm operation

The obtained undiluted AF having pH 10.5 was further used as a sanitizer in the regular farm operation in a shed (G1) where the control shed (G2) was followed accordingly, and tap water was used instead of AF. The mortality rate was reduced by approximately half in the AF treatment (4.76%) shed compared to the control untreated (9.52%) shed. The higher body weight gain, low feed conversion (FCR) ratio, and low mortality were noticed for G1 in comparison to G2 (Table 4).

## Discussion

In Bangladesh, household ashes are the residual product commonly produced by rural people during cooking as a fuel source or cleaning as a sanitizer. Previously used ash as a hand sanitizer in post-defecation in a particular area in Bangladesh reduced diarrheal pathogen transmission through contaminated hands practiced also in Kolkata, India, among rural and slum people for hand washing [27]. In a cost-effective water purification filtration technique, rice husk ash worked as the base material for tapping 95% turbidity with bacteria and flies, where ash is a source of activated carbon that can catch the organic matter [28].

**Table 4.** The number of dead birds, mortality rates, body weights, and FCR ratio of AF treated broiler shed and PBS treated control shed. Both the sheds were managed with proper biosecurity care.

Parameter	No. of birds	No. of dead birds	Mortality rate	Body weight gained (32nd day)	Total feed consumed	FCR
AF treated birds (G1)	210	10	4.76	1,880 g/bird	2,738.09 g/bird	1.45%
Control Birds (G2)	210	20	9.52	1,810 g/bird	2,738.09 g/bird	1.51%

However, the antimicrobial efficacy of AF from household ashes has not yet been addressed. Therefore, the current study aimed to prepare the AF from household-derived ashes using an in-house-designed handmade filtration system with the estimation of their pH as an indicator of their alkaline state, analysis of the chemical constituents of pooled AF, investigation of the antimicrobial activity against *Salmonella* spp., LPAIV H_9_N_2_, and NDV, and finally as an experimental sanitizer substitute to chemical disinfectant for sanitizing eggshells as well as in poultry farm operation as a part of a biosecurity program.

In this study, out of 20 collected household-derived ashes from various plant sources (straws, leaves, woods, roots, and so on), after adding water, five prepared AF showed high alkaline pH 10.5–10.00 originating from rice straw, plum woods, neem wood, mahogany wood, and mango wood, using a handmade filtration system that consists of five chambers, which produced clear and colorless AF. In the upgraded filtration system, cotton and small and large stones were used in addition to sand, charcoal, and foam. A previous study suggested the use of cotton in Whatman filter paper to filter urine samples for hormone analysis showed better efficacy [29]. In the current study, ash from different plant origins showed variation in the pH, as it has been described that the physical and chemical features of ash depend upon the type of tree, part of the tree, geographic location, method, and temperature of combustion [14,16], as well as on the filtration system. Therefore, the mineral content and pH of the AF can be different. Wood ash contains alkali metal and alkaline earth elements in the form of oxides, hydroxides, and carbonates, which are mainly responsible for their high alkalinity [15]. Several studies reported that the alkali contents of the ash-derived potash were mainly carbonates of potassium and sodium [30].

Chemical analysis of the pooled AF at pH 11 to 10.5 revealed a very high concentration of K and Na with the presence of HCO_3_- and CO_3_-, causing high alkalinity of the AF. Moreover, Cl concentration was also high in pool AF, which may be responsible for disinfectant activity; a study suggested that in the form of dioxide, Cl was effective against the resistant Mycobacterium, H_1_N_1_, and other influenza viruses [31]. Other components, Ca, Mg, P, and S, were present in pools of AF with moderate to lower concentrations. Moreover, several studies reported that ash-derived alkali from various sources such as palm bunch, cassava peels, plantain peels, and agro waste are used as raw materials for making various kinds of soap [19,30–32]. Similarly, the obtained AF, having a similar lathering appearance to soft soap with a highly alkaline nature, can be substituted for other chemical soap, detergent, or disinfectant.

Before antibacterial efficacy testing, the study isolated *Salmonella* spp. from market table eggshell surface samples. *Salmonella* contamination was found in 11% of cases, close to the previous findings of 13.3% on eggshells at different markets in Dhaka city [10]. Next, from the antibacterial efficacy testing, *Salmonella* treated with undiluted and diluted AF of pH >10.0 gradually inhibited the growth. Significant inhibition was also observed in positive control washing soda and sodium hydroxide at pH 11, whereas the negative control NB and DW showed the usual growth of the bacteria, as *Salmonella* spp. can grow in an optimum pH range of 7–7.5 [23,27]. The high alkalinity of the AF is responsible for the inhibition of *Salmonella* growth; thus, AF remains a potent bactericidal agent against *Salmonella *spp.

Previous research focused on the antimicrobial activity of ashes from other sources, such as *Punica granatum* L. fruit peel ashes against *Candida albicans*, *Escherichia coli*, *Pseudomonas aeruginosa*, *Salmonella enterica* serotype *Typhimirium*, and *Shigellaflexneri*, evaporated extract of cow dung ash at basic pH 11.7 against *Cyanobacteria*, *Staphylococcus aureus*, *Bacillus subtilis*, and *E. coli* using different analytical techniques extract of dried Kadali banana peel powder ash against *Aspergillus niger* [33,34] are in line with the current antibacterial efficacy against *Salmonella *spp.

In the case of an antiviral efficacy trial, both undiluted and different diluted AFs at pH ranging from 10.5 to 9.8 sprayed on NDV and LPAIV were unable to inactivate the virus completely, but HA titer in the allantoic fluid was comparatively lower than inoculation of untreated and DW-treated virus, suggesting a reduction of viral growth by AF treatment (Fig. 5). H_9_N_2_ virus treated with undiluted and different diluted AF from pH range 10.0–10.5 resulted in lower HA titer detected in the allantoic fluid after embryo inoculation than the NDV (Fig. 5A and B). Both NDV and H_9_N_2_ viruses, individually treated with washing soda and sodium hydroxide, caused complete inactivation of the virus, as the HA titer was zero in the allantoic fluid, whereas untreated and DW-treated viruses showed comparatively higher HA titer in allantoic fluid than AF-treated viruses. AF up to pH 10.5 was not efficient for completing inactivation of the velogenic strain of ND virus used in this experiment; thus, a more alkaline AF pH >10.5 was needed to complete inactivation. However, the LPAIV (H_9_N_2_) is somewhat sensitive to AF from pH 10.5 and achieves comparatively lower virus growth. Researchers said that the lipid envelope of the AIV is mainly responsible for being easily susceptible to all types of disinfectants [31,33].

Before marketing, eggs should be properly sanitized to avoid hazards associated with contaminated eggshells. Before human consumption, natural plant extract disinfects the table and eggshell using 1% licorice plant extract immersed for 5 min, resulting in complete decontamination of eggshells that were experimentally contaminated with *S. typhimurium* [12]. Since using chemical disinfectant as an egg sanitizer causes a residual effect on public health and developing resistance against pathogens, in our study, we applied our AF of pH 10.5 on the eggshell surfaces to decontaminate eggs from microbial burden. Untreated eggs showed comparatively higher densities of bacterial count (6 × 10^3^ CFU/ml) than AF-dipped (0.63 × 102 CFU/ml) and sprayed (0.23 × 102 CFU/ml) on the eggshell surface, where AF-dipped treatment exposed the best result in the reduction of bacterial count. Moreover, DW-dipped and sprayed treatment did not achieve satisfactory bacterial count reduction. Disinfectants become resistant due to repeated and wrong use [34]. Substituting the chemical disinfectant, we further used undiluted AF with pH 10.5 as a sanitizer during the cleaning and disinfection procedure of an experimental broiler shed, resulting in a reduction of bird mortality by approximately half time compared to another control shed where normal water was being used. Moreover, higher body weight gain, low, and low mortality rate were noticed for the AF-treated flock compared to the control flock. Ideally, AF can be used as a natural, easily available, cost-effective sanitizing agent substitute for other chemical disinfectants in farm operations. However, the antimicrobial efficacy of AF against other common disease-causing bacterial, viral, and fungal pathogens with a broad range should be required. A further large-scale study is essential for the commercial farm application of AF in their routine biosecurity practice.

## Conclusion

Widespread use of chemical disinfectants in poultry farm biosecurity practices results in antimicrobial resistance and creates environmental, animal, and human health hazards. In our study, as a substitute for chemical disinfectant, we prepared AF from household ashes through a hand-made filtration system that mostly carried a high alkaline pH ≥10 and was rich in K, Na, and Cl. Our study revealed that the AF of pH >10.5 and its dilutions gradually inhibit *Salmonella *growth and show pH-dependent antibacterial activity. AF successfully worked as an egg sanitizing agent; thus, both AF sprayed and dipped reduced significantly the bacterial loads on the eggshell surface compared to the untreated eggs. On the other hand, AF up to pH 10.5 did not show optimum antiviral efficacy against the velogenic strain of NDV but somewhat showed lower growth against the LPAIV (H_9_N_2_). Further using AF with pH 10.5 as a routine sanitizer in the biosecurity program of an experimental boiler shed achieved low bird mortality, higher body weight gain, and a low compared to the control flock. Since AF showed antimicrobial efficacy, it could be a good choice for low-income farmers to use as a substitute for commercial egg sanitizer or disinfectant in their farm operation, which is natural and cost-effective.

## List of abbreviations

AF, Ash filtrate; CFU, Colony forming unit; DW, Distilled water; FCR, Feed conversion ratio; NaOH, Sodium hydroxide; NDV, Newcastle disease virus; LPAIV, Low pathogenic avian influenza virus; WS, Washing soda.
